# Unveiling Multi-Hazard Hotspots in Kabul: An Integrated Geospatial Assessment in a Data-Scarce Urban Environment

**DOI:** 10.1007/s00267-026-02588-w

**Published:** 2026-09-03

**Authors:** Maisam Rafiee, Mohammad Reza Ansari, Daniela Kempa, Tinka Kuhn, Blal Adem Esmail, Lisa Biber-Freudenberger, Christian Albert

**Affiliations:** 1 Institute of Environmental Planning, Leibniz University Hannover, Hannover, Germany; 2 Institute of Geography, Ruhr University Bochum, Bochum, Germany; 3 GLOMOS - Center for Global Mountain Safeguard Research, Eurac Research, Bolzano, Italy; 4 Center for Development Research, University of Bonn, Bonn, Germany; 5 Institute of Crop Science and Resource Conservation, Bonn, Germany

**Keywords:** Multi-criteria approach, Multi-hazard mapping, Environmental hazard assessment, Spatial distribution, Kabul, Afghanistan

## Abstract

Multi-hazard assessment plays a crucial role in understanding the spatial concurrence and cumulative effects of various environmental hazards. However, limited availability of integrated hazard information and the lack of spatially explicit multi-hazard assessments constrain urban risk governance and landscape-scale nature-based solution planning. This study addresses this gap by enhancing scientific understanding of the spatial distribution of multiple environmental hazards in Kabul region, where data limitations and informal growth hinder effective risk management. The study pursues two objectives: (1) to assess and map the spatial distribution of individual environmental hazards, and (2) to identify multi-hazard zones and their specific hazard combinations. This study assesses five key hazards—flood (FH), heat (HH), drought (DH), soil erosion (SEH), and groundwater stress (GWS)—using Spatial Multi-Criteria Decision Analysis within a GIS framework. The study rates and weights nine parameters through hazard-specific methods. It then integrates the binary hazard layers using two approaches: (1) raster calculator to quantify the spatial overlap of hazard-prone areas, and (2) weighted multiplication of binary layers to interpret unique hazard combinations. The results show that approximately 80% of Kabul experiences at least three hazards. Single-hazard zones account for 1.8% of the study area, while two-, three-, four-, and five-hazard zones represent 18.6%, 44.3%, 32.9%, and 2.8%, respectively. FH emerged as the most spatially extensive individual hazard (8.5%), with frequent combinations including FH–GWS (8.47%), GWS–HH (17.79%), SEH–HH (8.54%), SEH–GWS–HH (18.81%), and DH–GWS–HH (8.16%). The findings enhance understanding of hazard combination and enable more targeted mitigation planning.

## Highlights

- Develops an integrative geospatial framework to map multiple environmental hazards in a data-scarce urban environment in the Global South.
- More than 80% of the study area is exposed to three or more concurrent hazards.
- Provides a hazard baseline to support urban risk governance and landscape-scale Nature-based Solutions (NBS) planning.

## Introduction

Urban areas across the globe are increasingly confronting a complex interplay of environmental hazards, with their frequency and intensity amplified by climate change, environmental degradation, and rapid urban expansion (Ranghieri et al., 2017; World Bank, 2021). These phenomena are not occurring in isolation; rather, they converge in intricate ways that challenge traditional urban planning and governance mechanisms. The stakes are particularly high in cities of the Global South, where rapid population growth and unregulated spatial expansion often outpace the development of adequate infrastructure and governance systems (Alikaei et al., 2023; Government of the Islamic Republic of Afghanistan, 2015). In such settings, risk-informed urban planning becomes more urgent and more difficult, especially where the availability of reliable environmental and spatial data is limited (Skilodimou et al., 2019; Alikaei et al., 2023; Morejón et al., 2025; Gohil et al., 2024).

Environmental hazards in urban areas are typically interlinked, both in their drivers and their consequences. As cities expand, they become increasingly susceptible to a range of natural and anthropogenic threats (Government of the Islamic Republic of Afghanistan, 2015; Alikaei et al., 2023; Ranghieri et al., 2017; Summers et al., 2021). Recent studies emphasize that environmental hazards rarely occur independently but often interact through cascading or compound processes. For example, heavy rainfall can simultaneously trigger flash floods, landslides, and debris flows, particularly in mountainous environments where topography and hydrological processes intensify hazard interactions (Ullah et al., 2022). Multi-hazard frameworks therefore provide a more realistic representation of disaster risk compared with single-hazard approaches, as they account for both individual hazards and their potential interactions (Hussain et al., 2025). Consequently, multi-hazard susceptibility mapping has become an increasingly important tool in disaster risk management and spatial planning. Such approaches enable the identification of areas exposed to multiple hazards simultaneously and provide critical spatial information for risk mitigation strategies and land-use planning (Rahman et al., 2024).

Nature-based Solutions (NBS), defined as actions that protect, sustainably manage, and restore ecosystems to address societal challenges, are increasingly promoted as integrated responses to hazard risk and climate adaptation (IUCN, 2020; Albert et al., 2019). Applications such as floodplain restoration, vegetated slope stabilization, and urban green corridors have demonstrated potential to reduce hazard exposure while generating co-benefits for biodiversity, climate resilience, and public well-being (Albert et al., 2019; IUCN, 2020; World Bank, 2021). Addressing such complex and dynamic challenges with NBS necessitates holistic and integrative planning approaches (i.e., combining hazard mapping with land-use planning). However, in many cities of the Global South, the effective planning and implementation of NBS is constrained by the lack of spatially explicit, science-based assessments of where hazards occur, how they interact, and which areas should be prioritized for intervention (Adem Esmail et al., 2022; Albert et al., 2021).

Scientific advancements in geospatial technologies offer unprecedented opportunities to bridge this knowledge-practice gap. Tools such as Geographic Information Systems (GIS), remote sensing, Multi-Criteria Decision Analysis (MCDA), and fuzzy logic modeling—have made it possible to work with fragmented or limited datasets to generate composite hazard assessments (Gohil et al., 2024; Skilodimou et al., 2019; Pourghasemi et al., 2019; Adem Esmail and Geneletti, 2018; Summers et al., 2021; Hussain et al., 2023; Al-Aizari et al., 2022).

Multi-hazard susceptibility mapping typically integrates environmental and topographic variables—including elevation, slope, rainfall, drainage density, land cover, geology, and proximity to rivers—because these factors strongly influence the spatial occurrence of floods, landslides, and related hazards (Rahman et al., 2024). These variables are commonly derived from remote sensing data and digital elevation models (DEMs), enabling spatial hazard modeling even in data-limited regions. Building on previous work in multi-hazard assessment (Sharma and Kumar Rana, 2024), normalized hazard layers can be overlaid to identify areas where specific hazard combinations coexist, thereby supporting the identification of multi-hazard hotspots that may potentially be addressed through NBS (Lopa et al., 2026; Sarker and Adnan, 2024; Sarker et al., 2026).

In addition, recent methodological developments highlight the growing role of machine learning and deep learning techniques in hazard prediction. For example, convolutional neural networks (CNNs) have been applied to multi-hazard susceptibility mapping to capture complex relationships between environmental variables and historical hazard events, often achieving higher predictive accuracy than traditional statistical models (Ullah et al., 2022). Similarly, hybrid modeling approaches that combine machine learning algorithms with GIS-based MCDA frameworks have demonstrated improved performance in predicting flood and multi-hazard susceptibility by integrating spatial environmental indicators with data-driven pattern recognition techniques (Hussain et al., 2023).

Despite the growing use of GIS-based MCDA for hazard assessment, its application in rapidly urbanizing mountainous cities of the Global South remains limited, particularly for planning-oriented multi-hazard analysis. In Kabul, existing assessments such as the World Bank’s Disaster Risk Profile for Afghanistan identify major hazards including floods, droughts, landslides, avalanches, and earthquakes at the national scale (World Bank, 2018; Ranghieri et al., 2017). However, these studies do not provide spatially explicit and integrative local-scale assessments that can directly support NBS planning. Urban planning in Kabul is further constrained by institutional fragmentation, inconsistent implementation of planning frameworks such as the Kabul Urban Design Framework (KUDF) (Sasaki, 2019), and the continued expansion of informal settlements into hazard-prone areas (Government of the Islamic Republic of Afghanistan, 2015). Existing research on Kabul’s hazard distribution also remains fragmented, with consultancy-based assessments and isolated hazard analyzes rarely incorporated into a scientifically integrated framework for urban resilience planning (Alikaei et al., 2023; Rahman et al., 2024).

Based on the environmental and geomorphological characteristics of the Kabul region, this study hypothesizes that environmental hazards exhibit distinct spatial patterns and that specific areas function as multi-hazard hotspots where several hazards converge. It is further hypothesized that geospatial multi-criteria analysis integrating environmental variables can effectively identify the spatial concurrence even in data-scarce contexts, thereby providing a reliable basis for guiding NBS planning.

This study addresses these gaps by developing a spatially explicit, multi-hazard assessment for the Kabul region, designed to identify hazard combinations that may be mitigated through NBS interventions in a data-scarce and hazard-prone environment. With the aim of enhancing scientific understanding of the spatial distribution and concurrence of multiple environmental hazards in the Kabul region, integrative mapping techniques are improved and adapted to data-scarce environments. The study is driven by two objectives: (1) to assess and map the spatial distribution of individual environmental hazards across Kabul city, and (2) to identify multi-hazard zones and specific hazard combinations through the spatial overlapping of individual hazards. The novelty of our approach lies in the spatially explicit and integrated multi-hazard assessment of the Kabul region, which combines multiple hazard susceptibility layers to identify overlapping hazard patterns and hazard combinations that can be addressed through nature-based solution (NBS) interventions in a data-scarce and hazard-prone environment. The analysis focuses on five hydro-climatic and environmental hazards—floods, droughts, soil erosion, heat, and groundwater stress— due to their strong linkages with environmental processes and their potential relevance for NBS; and applies a geospatial methodology integrating remote sensing, environmental variables, and MCDA (Sharma and Kumar Rana, 2024; Kundu et al., 2021; Gohil et al., 2024). Seismic hazards such as earthquakes were excluded because they require fundamentally different assessment frameworks, indicators, and mitigation approaches beyond the scope of this study. The MCDA approach is used in this study because it allows for the systematic integration of multiple, diverse environmental and physiographic factors that contribute to different hazard processes, even when there are no clear quantitative relationships between them. MCDA provides a transparent, structured, and replicable framework for incorporating expert knowledge and empirical evidence when weighting and combining these parameters for hazard assessment (Nascimento et al., 2017; Saaty, 2008). In this study, the weights of the parameters for all hazards except the heat hazard—which have been assessed through land surface temperature (LST) modeling (Khan et al., 2022)—were estimated using the multi-criteria Analytical Hierarchy Process (AHP) method developed by Saaty (2008) within a GIS environment (Gohil et al., 2024; Sharma and Kumar Rana, 2024; Kundu et al., 2021). This study contributes to urban risk governance by delivering a comprehensive spatial assessment of five major environmental hazards in Kabul, using geospatial and multi-criteria tools to identify both individual and overlapping hazard zones. These insights enhance the understanding of hazard combinations and enable more targeted mitigation planning. Although the study does not assess population exposure or socio-economic vulnerability, it provides a critical hazard baseline for guiding NBS. As such, it marks a foundational step toward operationalizing data-informed, landscape-scale planning in data-scarce urban contexts like Kabul.

## Methodology

### Study Area

**Fig. 1:**
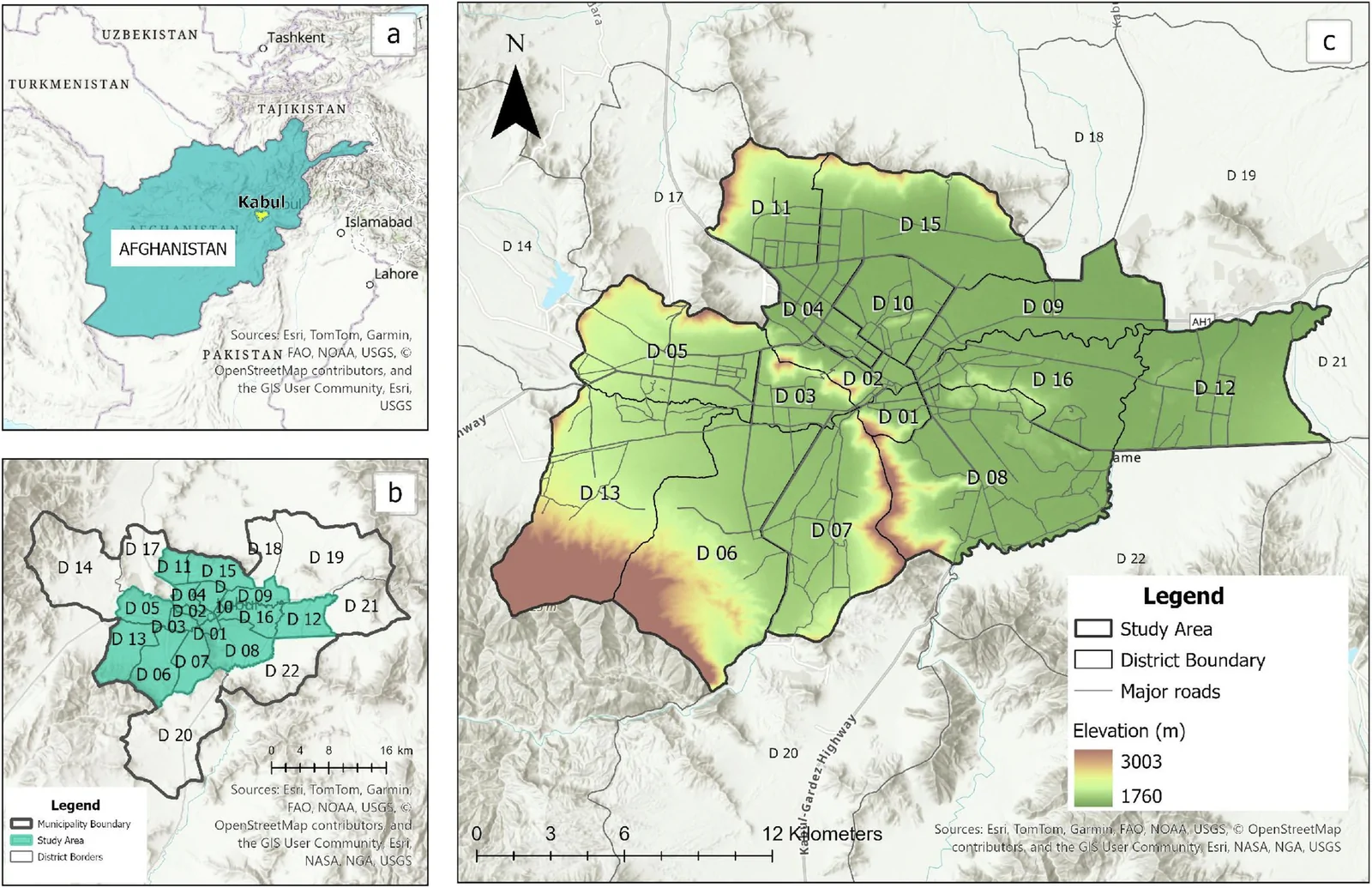
**a** Location of Afghanistan, **b** Kabul Municipality Boundary, **c** Study Area (D01-D22 corresponds to Districts 1-22)

Kabul city, the capital of Afghanistan (see Fig. 1), is located in the central-eastern part of the country within the Kabul plain at an elevation of approximately 1.800  m above sea level (Zaryab et al., 2022a; Mushwani et al., 2024a). The city’s landscape is ecologically diverse, with snowmelt from the mountains to the west sustaining seasonal rivers, and fertile agricultural lands located to the east (Sasaki, 2019). Kabul experiences a highly seasonal climate, with wet winters and springs followed by hot, dry summers and falls. These climatic dynamics, combined with its rugged topography and accelerated urban growth, make the city increasingly vulnerable to a variety of environmental hazards (Sasaki, 2019; Zaryab et al., 2022a; Ranghieri et al., 2017).

Kabul faces significant exposure to hydrometeorological hazards including flash floods, droughts, soil erosion, extreme heat, and groundwater stress. Flooding, driven by intense precipitation and snowmelt, is the most frequently occurring hazard and is worsened by outdated and insufficient drainage systems (Ranghieri et al., 2017; Mushwani et al., 2024b). Droughts are recurring and intensifying, leading to diminished agricultural productivity and strained water resources (Ranghieri et al., 2017). The city also experiences growing heat stress, amplified by extensive impervious surfaces and minimal vegetative cover. Soil erosion on the city’s steep, deforested hillsides further increases environmental risks, especially in informal settlements. In parallel, Kabul’s groundwater resources are under acute pressure. The city depends almost entirely on two shallow Quaternary aquifers—the central Kabul and Paghman-Darulaman aquifers—which have been extensively overexploited. Groundwater levels have declined by up to 60 meters in some areas, and water quality is increasingly compromised due to contamination and saline intrusion (Zaryab et al., 2022a; Brati et al., 2019).

Kabul’s urban population has surged from around one million in 2001 to over 5.3 million in 2021, making it one of the fastest-growing cities globally (Zaryab et al., 2022a). A significant share of the population—more than 70%—resides in unplanned or informal settlements, with particularly high densities in municipal districts D01- D13, D15 and D16 (Fig. 1) (Sasaki, 2019; Maisam, 2018). This unregulated growth has resulted in widespread land-use change, notably a 40% increase in built-up areas and a 32% reduction in agricultural land between 2000 and 2020 (Zaryab et al., 2022a). These changes have intensified pressure on the urban environment and infrastructure, exacerbating vulnerability to environmental hazards.

### Material

**Fig. 2:**
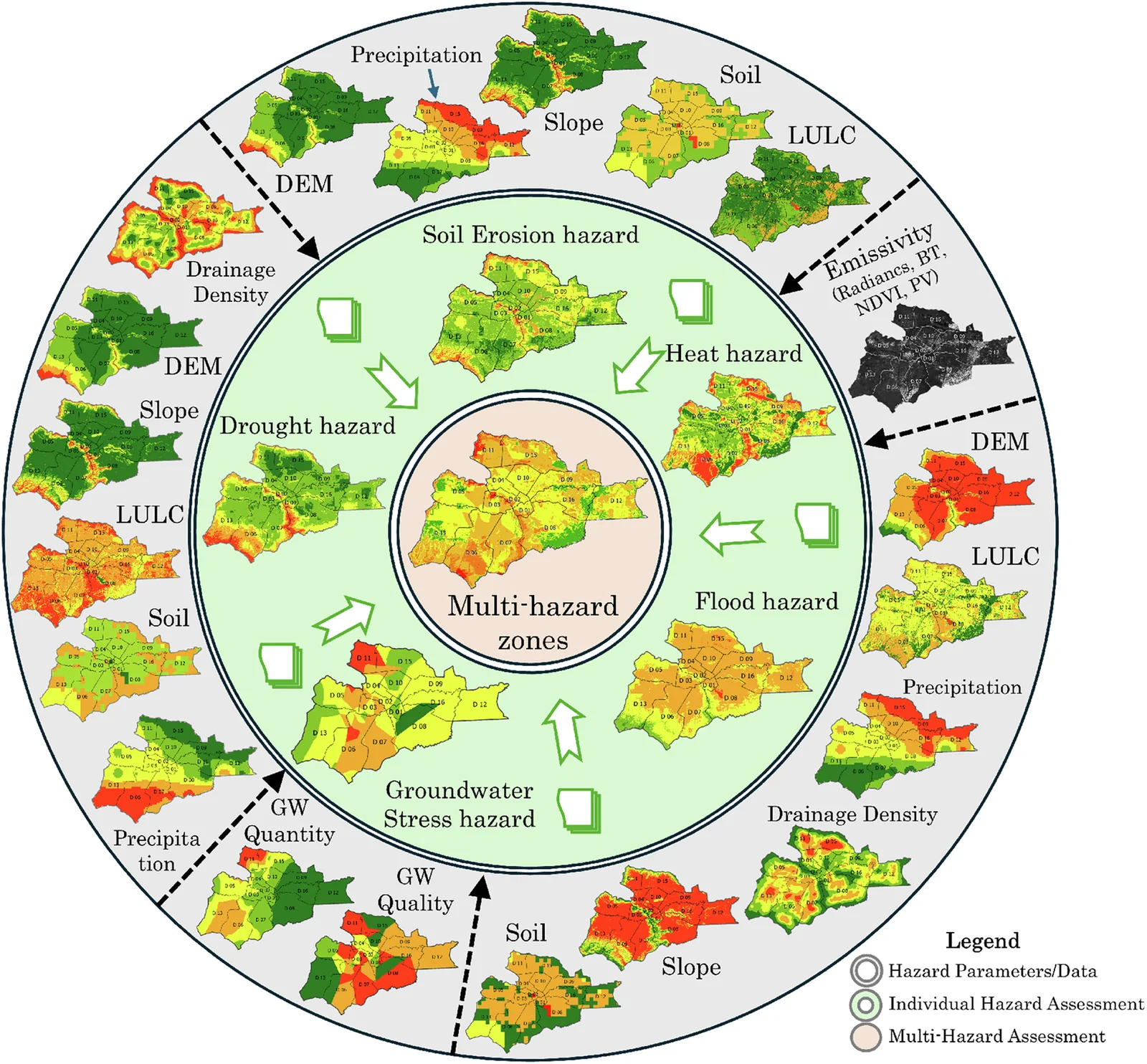
Methodological framework: the outer ring of the framework delineates the parameters selected for the assessment of each individual hazard. The middle ring of the framework illustrates the individual hazard assessment results. The central circle of the framework illustrates the results of multi-hazard zones and combinations. (DEM Digital Elevation Model, LULC Land Use Land Cover, GW Groundwater, BT Brightness Temperature, NDVI Normalized Difference Vegetation Index, PV Proportion of Vegetation)

The data employed in this study primarily comprises Landsat 8 imagery, which was utilized to generate LST (Khan et al., 2022). Land Use and Land Cover (LULC) data were derived by integrating two global datasets: Esri Sentinel-2 and OSM Geofabrik (see Table 1). An accuracy assessment of the resulting classification yielded a Kappa coefficient of 0.85, indicating a high level of agreement (see Table 4 for the confusion matrix in the Online Resource). As a result, the LULC of the study area was categorized into five distinct classes: Agricultural Land, Vegetation Cover, Built-up Areas, Bare Land, and Water Bodies. These classifications were integral to the assessment of flood, soil erosion, and drought hazards. The Digital Elevation Model (DEM, hereinafter elevation) was classified into five distinct categories using the natural breaks (Jenks) method (Gohil et al., 2024). The slope layer was derived from the elevation layer of the study area and classified into five categories using the natural breaks method, as well as being generated through a resampling technique, specifically by the bilinear interpolation method with a cell size of 30 × 30 (Gohil et al., 2024; Skilodimou et al., 2019). The drainage density was derived using an elevation layer and a well-defined methodology outlined by Chandrappa et al. (2023). The resulting drainage density values were classified into five categories using the natural breaks method (Jenks). Precipitation data served as a critical parameter in this study, playing a pivotal role in evaluating flood, soil erosion, and drought hazards. The average of annual precipitation over a 10-year period (2013–2022) from two data sources (CHIRPS v3.0 and PERSIANN-CCS) was analyzed using the Inverse Distance Weighting (IDW) interpolation method to generate continuous precipitation surfaces. Subsequently, the interpolated outputs from both datasets were averaged to produce the final precipitation map used in the analysis. IDW was selected because it assumes that nearby observations exert greater influence on estimated values than distant observations, which is appropriate for representing spatial variability in precipitation patterns within the study area. Furthermore, given the limited and uneven distribution of available precipitation observations, IDW provides a straightforward and widely applied interpolation approach that does not require assumptions regarding spatial autocorrelation structures or variogram modeling, as required by more complex geostatistical techniques such as kriging (Skilodimou et al., 2019; Setianto and Triandini, 2013). The map classification is performed through the natural breaks method (Jenks) (Skilodimou et al., 2019). Furthermore, the study area is predominantly characterized by five distinct soil types: Arenosols, Nitisols, Solonetz, Glaciers, and open water. The soil data layer was resampled using the nearest neighbor method in ArcGIS Pro 3.4.3, with a cell size of 30 × 30 m (Harmonized World Soil Database version 2.0, 2023). Nitrate concentration data were analyzed to develop a groundwater quality map (Brati et al., 2019; Shamsudduha et al., 2020), while groundwater depletion and depth data were used to create a groundwater quantity map (Shamsudduha et al., 2020; Zaryab et al., 2022a). The groundwater stress hazard assessment was derived from the integration of groundwater depletion and depth data, providing a comprehensive evaluation of groundwater stress hazards (Shamsudduha et al., 2020). Last but not least, the datasets originate from different sources and spatial resolutions; hence, all raster layers were resampled to a common spatial resolution of 30 m to ensure spatial consistency in the analysis. Point-based groundwater datasets were interpolated to continuous raster surfaces using GIS-based spatial interpolation methods prior to integration into the multi-hazard susceptibility mapping. Table 1 shows the details of required data collected through various sources for the assessment of individual hazards and multi-hazards; Fig. 2 illustrates the overall study framework and presents the different assessment levels in a concise manner.

**Table 1:** Primary data used in this study

Data	Resolution/ scale	Year	Source	Applicability
Landsat 8 image	30 m	2020	https://earthexplorer.usgs.gov/	For heat hazard
Esri Sentinel2	10 m	2020	https://livingatlas.arcgis.com/landcoverexplorer/#mapCenter=7.26596%2C52.50859%2C8.93&mode=step&timeExtent=2017%2C2024&year=2021	For flood hazard, soil erosion hazard and drought hazards
OSM Geofabrik LULC	shp	2025	http://download.geofabrik.de/asia/afghanistan.html	For flood hazard, soil erosion hazard and drought hazards
Elevation (DEM)	30 m	2013	Shuttle Radar Topography Mission (SRTM GL1) https://opentopography.org/	For flood hazard, soil erosion hazard and drought hazards
Precipitation	4 km × 4 km	2013–2022	https://chrsdata.eng.uci.edu/	For flood hazard, soil erosion hazard and drought hazards
	0.05 degree	2013–2022	https://chc.ucsb.edu/data/chirps3	For flood hazard, soil erosion hazard and drought hazards
Soil type	1 km × 1 km	2023	FAO & IIASA. https://doi.org/10.4060/cc3823en	For flood hazard, soil erosion hazard and drought hazards
Nitrate concentration	Point data	2019	Brati et al. (2019)	For groundwater stress hazard
Groundwater depletion	Point data	2007–2020	Zaryab et al. (2022b)	For groundwater stress hazard
Groundwater depth	Point data	2017	Jawadi et al. (2024)	For groundwater stress hazard

### Individual Hazard Assessment

#### Flood Hazard Assessment

The Analytic Hierarchy Process (AHP) method, recognized for its ability to yield accurate and reliable predictions, has been widely utilized in the delineation of flood-prone areas (Mushwani et al., 2024b). The flood hazard assessment in the current study was conducted based on the parameters established by Kourgialas and Karatzas (2011), with modifications introduced by Mushwani et al. (2024b). Considering the primary focus of this study, which is hazard assessment, certain modifications were made to the AHP method and its weightings, which have been considered by Mushwani et al. (2024b); in particular, flow accumulation was replaced with drainage density, as the latter has been identified as a critical parameter in flood hazard assessment by Gohil et al. (2024). Additionally, population density, which was initially considered an exposure factor, was excluded from the analysis. Thus, the selected parameters in this study are elevation, slope, soil type, drainage density, precipitation and LULC. The weights of each parameter are estimated through the multi-criteria AHP method, which is developed by Saaty in 1980 and employed in a GIS environment (Sharma and Kumar Rana, 2024; Kundu et al., 2021; Gohil et al., 2024). The DEM serves as one of the fundamental datasets. It is critical for deriving flood-related parameters, such as elevation, slope, the direction of runoff movement, the extent of inundation, and the water depth during flood events (Gohil et al., 2024; Skilodimou et al., 2019; Mushwani et al., 2024b). It is evident that gentle slopes contribute to favorable conditions for flooding (Mushwani et al., 2024b; Skilodimou et al., 2019; Gohil et al., 2024); thus, the highest flood rates were assigned to areas with the lowest slope. The drainage density is also one of the important parameters in flood hazard assessment; the highest rate was assigned to areas with a drainage density of 1.89 km/km^2^ or higher. The soil types were classified and ranked based on methodologies established in prior literature on flood hazard mapping (Mushwani et al., 2024b). A common component in flood hazard is precipitation, whereas heavy rainfall causes flooding (Gohil et al., 2024). The classification ratings for LULC categories were primarily determined based on methodologies outlined in two key studies: Gohil et al. (2024) and Mushwani et al. (2024b), Specifically, the lowest flood rating was assigned to vegetation cover and Agricultural Land due to its reduced contribution to surface runoff, while the highest ratings were allocated to Water Bodies and Bare Land, classified as very high and high, respectively. Table 2 shows the estimated weight of flood parameters and acceptable consistency ratio (less than 0.1); moreover, the classification, rating, pairwise comparison scores, and weight estimation of all parameters, along with their corresponding thematic maps, are provided in the Online Resource (Table 5a–c, and Fig. 8).

**Table 2:** Normalized weights and the consistency ratio of each parameter of selected hazards

Hazards	Parameters	Weights	Lambda	Consistency Index (CI)	Random Consistency Index (RCI)	Consistency ratio (CR)
Flood	Slope	0.11	6.2651	0.1029	1.24	0.08295
	Drainage Density	0.24	6.5083			
	Precipitation	0.18	6.4697			
	Soil type	0.08	6.1354			
	LULC	0.05	6.1836			
	Elevation	0.33	6.5143			
Soil erosion	LULC	0.08	5.0620	0.1077	1.12	0.09612
	Soil type	0.16	5.2295			
	Precipitation	0.41	5.2928			
	Slope	0.29	5.4306			
	Elevation	0.07	5.1518			
Drought	Slope	0.07	6.1248	0.1193	1.24	0.09624
	Drainage Density	0.24	6.5967			
	Precipitation	0.18	6.4666			
	Soil type	0.10	6.3148			
	LULC	0.06	6.1879			
	Elevation	0.36	6.4775			
Groundwater stress	GW Depletion	0.11	3.0159	0.0570	0.58	0.09828
	GW Depth	0.18	3.0327			
	GW Quality	0.70	3.1140			

#### Heat Hazard Assessment

Land Surface Temperature (LST) is a widely recognized proxy for surface thermal conditions and serves as a valuable indicator of thermal hazard, particularly in densely populated urban environments (Cheval et al., 2023). For this assessment, Landsat 8 imagery acquired on June 18, 2020—capturing only daytime temperatures—was utilized to identify spatial patterns of extreme surface heat (Cheval et al., 2023). The Landsat image was selected from the available imagery for June 2020, as this year recorded the highest temperature within the study period (Weather and Climate 2020). A cloud cover threshold of less than 30% was applied, excluding images with unknown cloud cover values by United States Geological Survey (USGS). Among the four available images acquired on 2, 11, 18, and 27 June, the image from 18 June was selected because it corresponded to the highest temperature day and exhibited a cloud cover value of 2.72%, which was the lowest cloud cover among all available images. LST retrieval was conducted using the approach described in the literature (Khan et al., 2022). The methodology involves a series of radiometric and spectral calculations applied to three key bands from Landsat 8: Thermal Infrared Sensor (TIRS) Band 10, Red Band 4, and Near-Infrared (NIR) Band 5. The process was implemented in ArcGIS Pro 3.4.3 using a custom-built model to automate the workflow (described in detail in the Online Resource “LST detailed process”).

Once the LST was computed, a binary heat hazard layer was created by identifying all areas where the LST exceeded a critical threshold of 40 °C. This threshold is consistent with literature standards and represents a significant level of thermal stress relevant to public health and urban climate studies (Cheval et al., 2023). Areas with LST values greater than 40 °C were classified as high hazard zones. The classification, along with thematic maps, is provided in the Online Resource (Table 6, and Fig. 9).

#### Drought Hazard Assessment

Assessing drought hazard is a critical step in evaluating the potential impacts of drought-related risks (Gohil et al., 2024). Drought hazard encompasses the physical characteristics of drought events, serving as a key factor in understanding the relationship between drought risk and regional susceptibility (Heydari Alamdarloo et al., 2020). This study adopts a spatial drought-susceptibility perspective, emphasizing the relative propensity of different areas to experience drought-related stress based on existing environmental characteristics rather than quantifying meteorological or hydrological drought events through temporal drought indices. Consequently, the assessment aims to identify areas that may be more susceptible to drought conditions given the current environmental context, rather than evaluating the frequency, duration, severity, or persistence of drought events. The assessment incorporates six parameters - slope, precipitation, soil type, LULC, elevation, and drainage density - which were selected based on established methodologies and findings in the existing literature to suit the context of drought hazard evaluation (Gohil et al., 2024). Subsequently, these parameters were integrated using the Raster Calculator tool in ArcGIS Pro 3.4.3. The weights of each parameter are estimated using the multi-criteria AHP method which is developed by Saaty in 1980 (Sharma and Kumar Rana, 2024; Kundu et al., 2021; Gohil et al., 2024). Table 2 shows the estimated weight of drought parameters and acceptable consistency ratio (less than 0.1); moreover, the classification, rating, pairwise comparison scores, and weight estimation of all parameters, along with their corresponding thematic maps, are provided in the Online Resource (Table 7a–c, and Fig. 10).

#### Groundwater Stress Hazard Assessment

The multi-parameter approach is guided by the methodology presented in Shamsudduha et al. (2020), who demonstrated the significance of jointly evaluating groundwater quality (e.g., arsenic, salinity) and quantity (e.g., storage depletion) for groundwater hazard mapping. The groundwater quality was assessed using nitrate (NO₃) concentration as a key indicator of contamination, particularly from anthropogenic sources such as sewage infiltration and agricultural runoff. Nitrate data were derived from point measurements documented in the study by Brati et al. (2019). These point data were spatially distributed using the Thiessen polygon method (Zaryab et al., 2022a) to delineate zones of influence for each sampling point. The resulting polygon-based layer was subsequently resampled to a 30 × 30 m resolution using the nearest neighbor technique in ArcGIS Pro 3.4.3. Groundwater depth data were sourced from Jawadi et al. (2024), and depletion trends were informed by the findings of Zaryab et al. (2022a). Both datasets were spatially interpolated using the kriging interpolation method with a spherical model, implemented via the Spatial Analyst tool in ArcGIS Pro 3.4.3. The three layers—groundwater depth and depletion and groundwater quality—were integrated using estimated weights obtained through the multi-criteria AHP method, which was developed by Saaty in 1980 (Sharma and Kumar Rana 2024; Kundu et al. 2021; Gohil et al. 2024). Table 2 shows the estimated weight of groundwater parameters and acceptable consistency ratio (less than 0.1); moreover, the classification, rating, pairwise comparison scores, and weight estimation of all parameters, along with their corresponding thematic maps, are provided in the Online Resource (Table 8a–c, and Fig. 11).

#### Soil Erosion Hazard Assessment

Soil erosion hazard assessment is essential for evaluating the spatial susceptibility of landscapes to soil degradation and sediment transport (Perera et al., 2020). Soil erosion is influenced by a combination of topographical, climatic, pedological, and land use factors, which together determine the degree of vulnerability in a given region (Perera et al., 2020; Gohil et al., 2024). In this study, soil erosion hazard is defined as the potential for soil loss driven by natural processes such as rainfall intensity, surface runoff, and slope dynamics. The assessment integrates five key parameters: slope, LULC, precipitation, soil type, and elevation. These parameters were selected and ranked in alignment with methodologies established in existing literature, particularly the approach presented by Gohil et al. (2024). The parameter layers were reclassified into ranked hazard scores as per standard susceptibility ratings derived from Gohil et al. (2024). The weights of each parameter are estimated through the multi-criteria AHP method, which was developed by Saaty in 1980 (Sharma and Kumar Rana, 2024; Kundu et al., 2021; Gohil et al., 2024). The Raster Calculator tool in ArcGIS was then used to weighted overlay of the reclassified layers and generate the final soil erosion hazard map. The resulting map was categorized into five classes: very low, low, moderate, high, and very high soil erosion hazard. Table 2 shows the estimated weight of soil erosion parameters and acceptable consistency ratio (less than 0.1); moreover, the classification, rating, pairwise comparison scores, and weight estimation of all parameters, along with their corresponding thematic maps, are provided in Online Resource (Table 9a–c, and Fig. 12).

### Multi-Hazard Assessment

Multi-hazard assessment was conducted by integrating individual hazard maps to analyze the spatial concurrence of multiple hazards across the study area. Initially, each individual hazard map—flood, drought, heat, groundwater stress, and soil erosion—was reclassified into binary categories using ArcGIS Pro 3.4.3. Specifically, areas identified as very low or low hazard were assigned a value of 0, while areas categorized as moderate, high or very high hazard were assigned a value of 1. This binary classification was applied to standardize the hazard layers prior to integration, as each individual hazard map was originally derived from different environmental variables and classification schemes (Effat and El-Zeiny, 2022; Kundu et al., 2021). Converting the maps into binary presence–absence categories allowed the analysis to focus on the spatial overlap of significant hazard zones, thereby avoiding bias caused by variations in hazard intensity scales across datasets (Effat and El-Zeiny, 2022). Furthermore, by considering moderate, high, and very high hazard classes as hazard presence, the analysis emphasizes areas with meaningful hazard levels for planning and mitigation purposes. Next, the reclassified hazard layers were combined using the Raster Calculator tool to compute the sum of the binary values for each pixel. This process yielded a composite raster with values ranging from 0 to 5, representing the number of overlapping hazard zones in a given pixel. The resulting map delineated zones with one hazard, two hazards, three hazards, four hazards, and all hazards, providing a spatial overview of hazard overlap intensity. In addition to the multi-hazard zones, a multi-hazard combinations assessment was generated to capture all unique combinations of the five individual hazards (Gohil et al., 2024). This was achieved by assigning a distinct numerical weight to each hazard layer (e.g., 1, 2, 4, 8, and 16) and multiplying each binary raster by its assigned weight (Eq. 1).

1 $${MH}={\sum }_{i=0}^{n}{w}_{i}\cdot {H}_{i}$$

Where:

*MH* = Multihazard combination index; *H*_*i*_ = Binary raster of hazard *i* (0 or 1); *w*_*i*_ = weight assigned to hazard I (Heat hazard=1, Groundwater stress=2, Soil erosion=4, Flood hazard=8, Drought hazard=16); *n* = number of hazards

The weighted rasters were then summed using the Raster Calculator, producing a map with 32 unique classes (2^5^ = 32), each representing a specific combination of hazard presence or absence. Finally, the attribute table of the resultant raster was interpreted to define the meaning of each unique class, indicating precisely which hazards were present in each combination.

## Results

### Individual Hazards Assessment

#### Flood Hazard Assessment

**Fig. 3:**
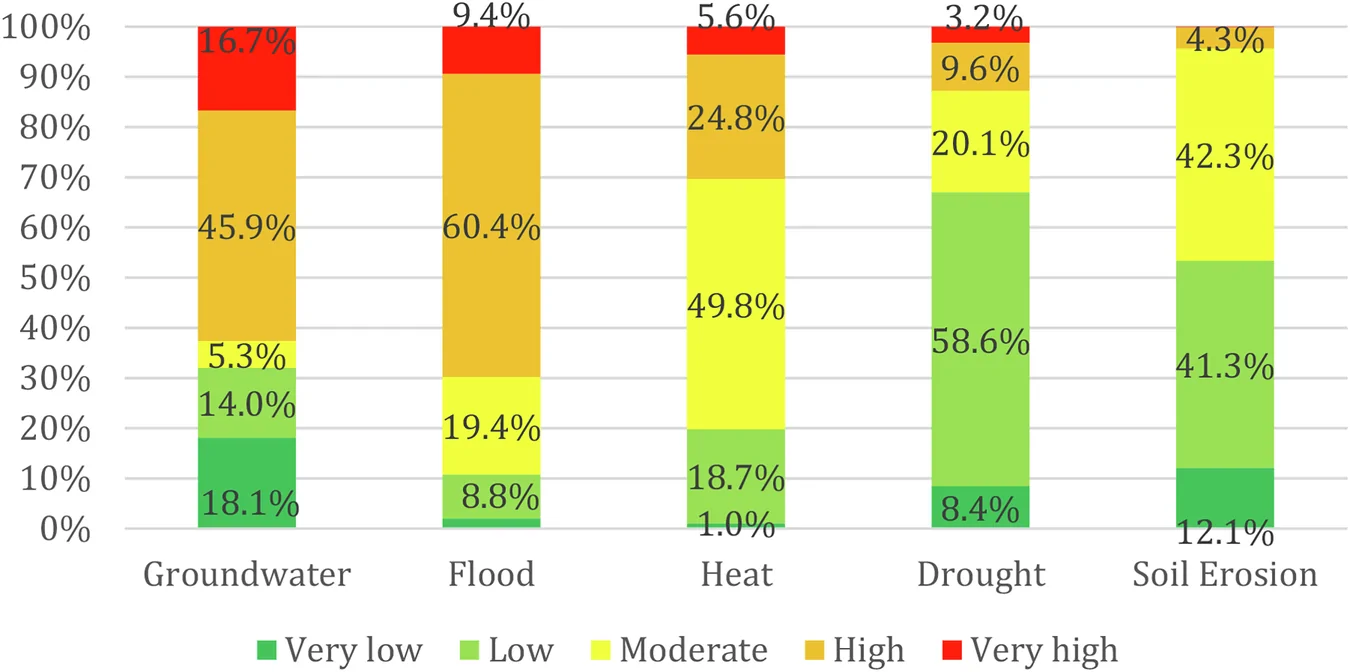
Spatial distribution percentages and corresponding classifications of individual hazards

**Fig. 4:**
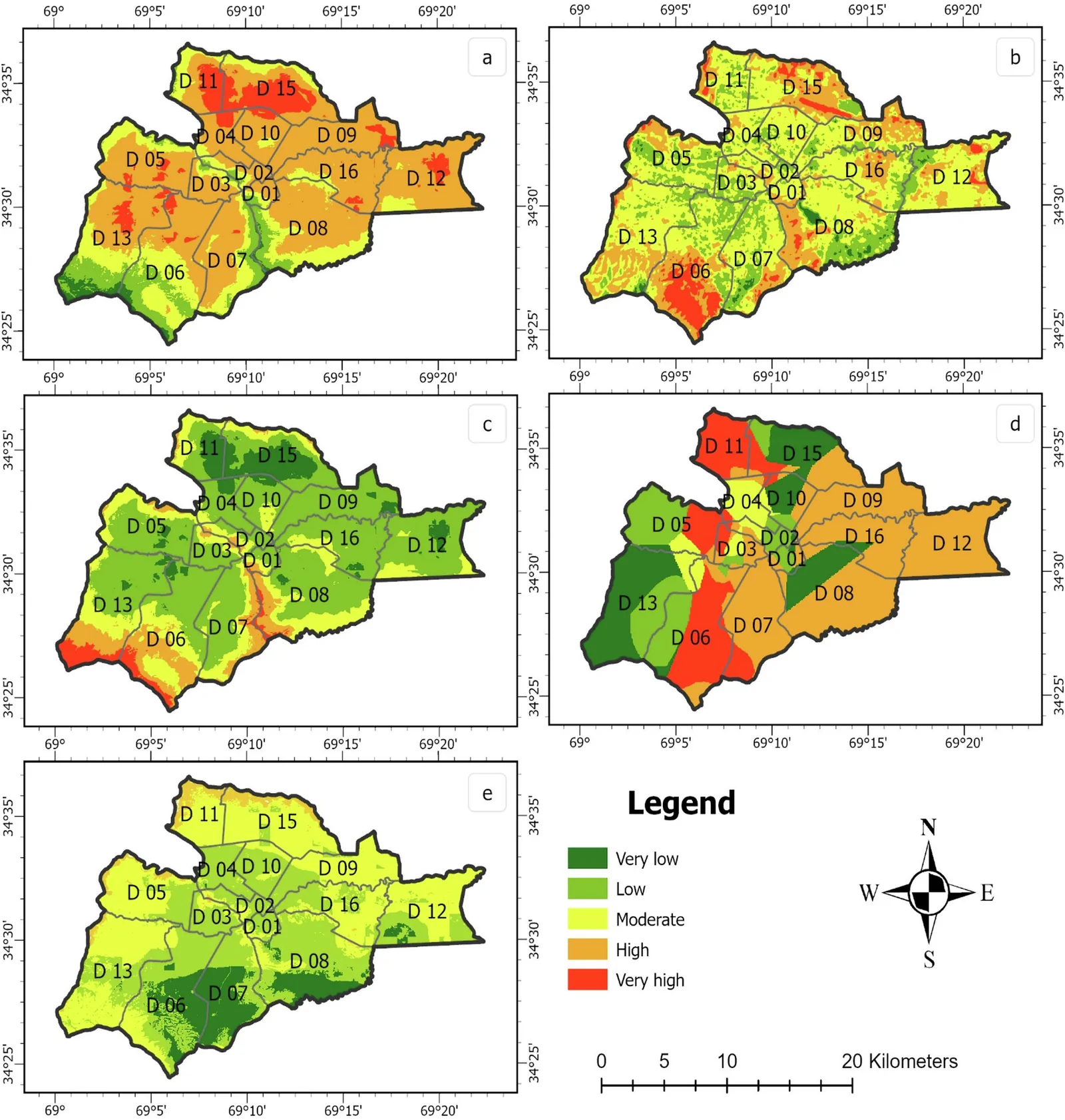
Individual hazards (D01-D22 corresponds to Districts 1-22), **a** Flood Hazard, **b** Heat Hazard, **c** Drought Hazard, **d** Groundwater Stress Hazard, **e** Soil Erosion Hazard

By integrating six key biophysical parameters—slope, drainage density, precipitation, soil type, land use/land cover (LULC), and elevation—the flood hazard (Fig. 4) provides a detailed understanding of the extent and distribution of flood-prone zones. The findings indicate that a significant portion of the study area, approximately 60 and 10% (Fig. 3), falls under the high and very high flood hazard categories, respectively. This widespread distribution of high and very high-hazard zones suggests a strong influence of unfavorable terrain characteristics, such as high precipitation, gentle slopes and low-lying elevations, which promote surface water accumulation. These zones were predominantly observed in the central and eastern districts (e.g., D01, D03, D07, D08, and D10), where urban development intersects with sensitive hydrological and geomorphological features. Furthermore, moderate hazard areas cover around 19% of the region, primarily distributed along transitional zones between high and low hazard districts. These areas are susceptible to flooding under extreme weather events, albeit to a lesser extent than the high-hazard zones. Although very high flood hazard zones account for a relatively small portion of the area (10%), their presence is particularly alarming due to their spatial concentration in densely built-up districts like D11 and D15, which are likely to host critical infrastructure and dense populations. In contrast, low hazard zones represent only 9% of the total area, mostly clustered in western districts such as D06 and D13. These zones typically feature higher elevations and steeper slopes, which facilitate rapid drainage and reduce flood susceptibility.

#### Heat Hazard Assessment

According to the results (Fig. 4), approximately 6% (Fig. 3) of the area falls under the Very High heat hazard category, primarily located in southern and northeastern urban districts such as D06, D08, D15, and parts of D07, D09 and D12. These zones are marked by extensive impervious surfaces, minimal vegetation, and dense built-up environments, which intensify the urban heat island (UHI) effect. An additional 24.8% of the area is classified under the High hazard category, indicating a significant spatial extent of thermal exposure across multiple districts. In contrast, Very Low and Low heat hazard zones account for 1% and 18.7% of the area, respectively. These districts, which include significant portions of D05, D03, D08, D07, and D11, benefit from higher levels of vegetative cover and natural land features that help mitigate surface temperatures. The Moderate hazard zone, which constitutes 49.8% of the study area, represents a transitional landscape with mixed land use and varying degrees of vegetation. These areas are at risk of escalating thermal exposure due to ongoing urban expansion and the potential degradation of existing green spaces.

#### Drought Hazards Assessment

The drought hazard assessment conducted in this study revealed substantial spatial variability in hazard levels across the study area. The findings indicate that the majority of the study area is characterized by low drought hazard, covering 58.6% (Fig. 3) of the total land surface. These zones (Fig. 4) are primarily situated in the central, eastern and northern parts of the region (e.g., D02, D04, D09, D10, D11, D12, and D16), where favorable topographic and hydrological conditions contribute to reduced hazard levels. Additionally, 8.4% of the area falls under the very low hazard category, bringing the total area with relatively low drought hazard to 67%. In contrast, moderate hazard zones account for 20.1% of the area and are typically located between low and high hazard regions. These areas represent transition zones that could become more susceptible over time, particularly under climate variability and land use changes. Notably, high and very high hazard zones constitute 9.6% and 3.2%, respectively. These areas are predominantly concentrated in the southern and southwestern districts (notably D06, D07, D08, and D13), where conditions such as steeper slopes, reduced precipitation, and limited vegetation cover increase vulnerability.

#### Groundwater Stress Hazard Assessment

The groundwater stress hazard assessment conducted in this study presents a spatially explicit analysis of groundwater-related hazards by integrating both groundwater quality and quantity indicators within a geospatial framework. This dual-dimensional approach, combining nitrate contamination levels and groundwater depth/depletion trends, provides a comprehensive perspective on aquifer vulnerability and sustainability across the study area. The resulting groundwater stress hazard (Fig. 4) reveals that a small portion of the urban region—5.3%—falls under the Moderate hazard category (Fig. 3). These areas, which span across central districts such as D03, D04, D05, D06, and D13, typically reflect a balance between moderate contamination levels and observable signs of groundwater depth/depletion. Although not yet critical, these zones are susceptible to escalating hazard levels under continued anthropogenic stress, such as urban expansion, agricultural intensification, and increased groundwater abstraction. In addition, 46% of the area is classified as experiencing High hazard, indicating a combination of pronounced groundwater stress and potential contamination risk. These zones are spatially concentrated in the eastern and southeastern districts (e.g., D07, D08, D09, D12, and D16), where urban land use patterns and declining water tables are likely to exacerbate both quality and quantity issues. Very High hazard zones constitute a share of 17% of the total area. Districts such as D11 and central parts of D06 show severe nitrate contamination and marked groundwater depth/depletion, posing significant challenges for safe water access and long-term aquifer viability. Conversely, Low and Very Low hazard areas collectively account for 32% of the region. These zones, observed primarily in the western and parts of central and northern districts such as D05 and D13, exhibit relatively favorable hydrogeological conditions with shallower, more stable groundwater levels and lower contamination hazards.

#### Soil Erosion Hazard Assessment

The largest proportion of the area falls under the very low and low hazard categories, accounting for approximately 53% of the total land area (See Figs. 3 and 4). This is followed by the moderate hazard class, representing 42.3%. In contrast, zones of high erosion hazard constitute 4.3%, indicating that while a majority of the region is not at immediate critical hazard, certain sub-areas exhibit significant susceptibility. The spatial distribution depicted in the hazard map further highlights that areas located in the northern peripheries—particularly within districts D11 and D15—exhibit concentrated zones of high erosion hazard. These zones are characterized by steeper slopes and possibly degraded or sparsely vegetated land cover, aligning with topographic and land use patterns known to exacerbate soil detachment and surface runoff. Conversely, a huge proportion of northern, eastern, western, and southwestern districts (such as D05, D06, D09, D11, D12, D13, D15, and D16) display a predominance of moderate hazard classes.

### Multi-Hazards Assessment

The individual hazard assessments presented in Section “Individual hazards assessment” revealed distinct spatial patterns across the study area, reflecting the influence of different environmental and anthropogenic drivers. Flood hazards are primarily concentrated in low-lying urban districts, while heat hazards are associated with densely built-up areas characterized by limited vegetation cover. In contrast, drought and soil erosion hazards are more prevalent in peripheral and elevated districts where precipitation is lower and vegetation cover is sparse. Groundwater stress shows a broader distribution across central urban districts due to combined pressures from groundwater abstraction and contamination risks. These differentiated spatial patterns provide the basis for the subsequent multi-hazard assessment, which examines how these individual hazards spatially overlap across the region.

**Fig. 5:**
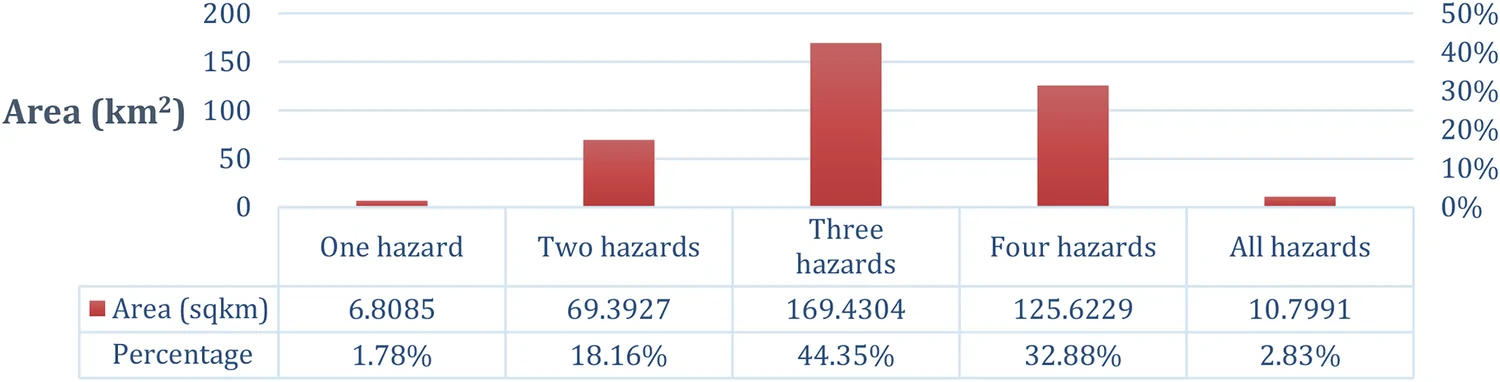
Spatial distribution of hazards’ zones

**Fig. 6:**
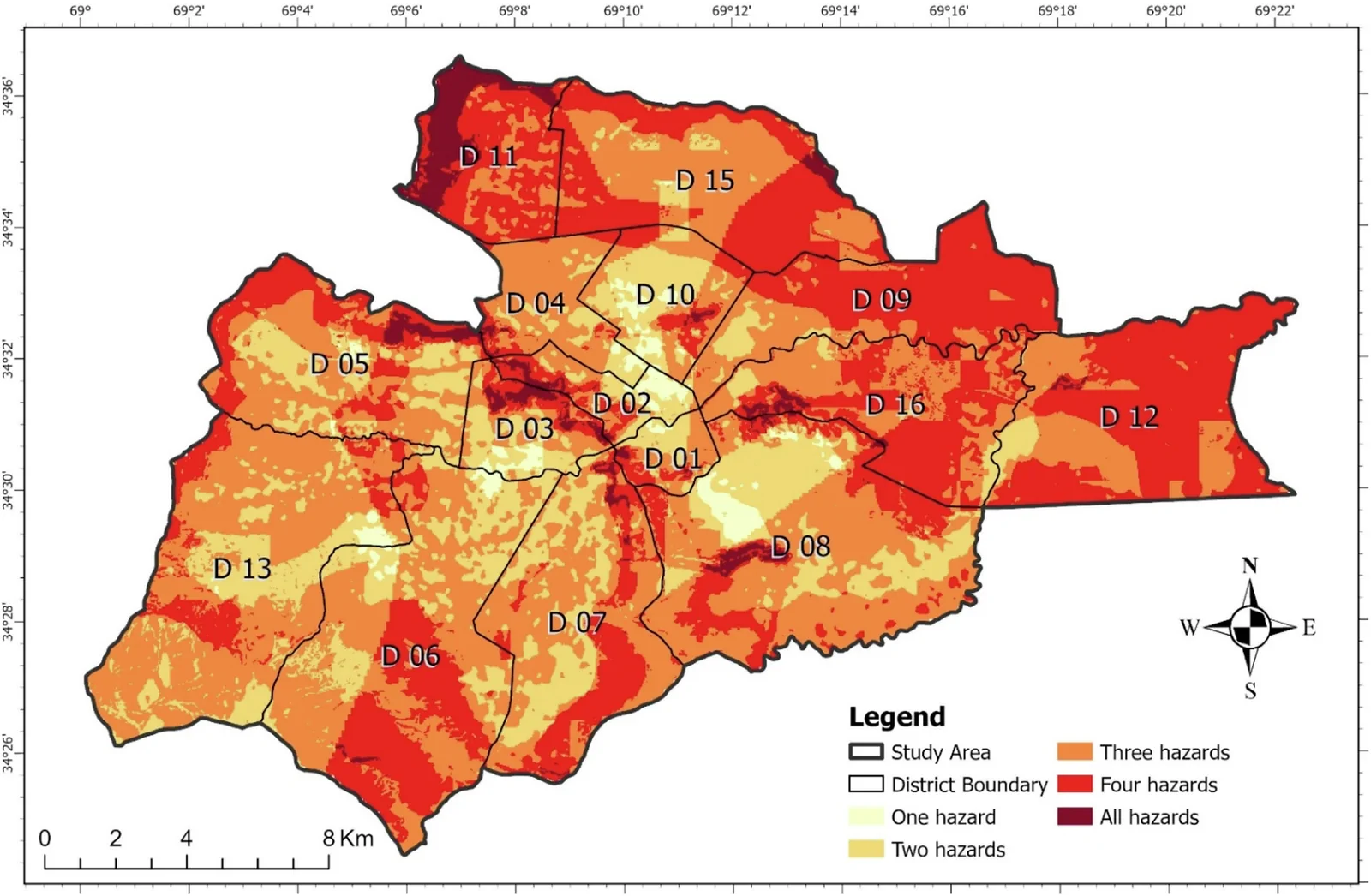
Multi-hazards zones (D01-D22 corresponds to Districts 1-22)

**Fig. 7:**
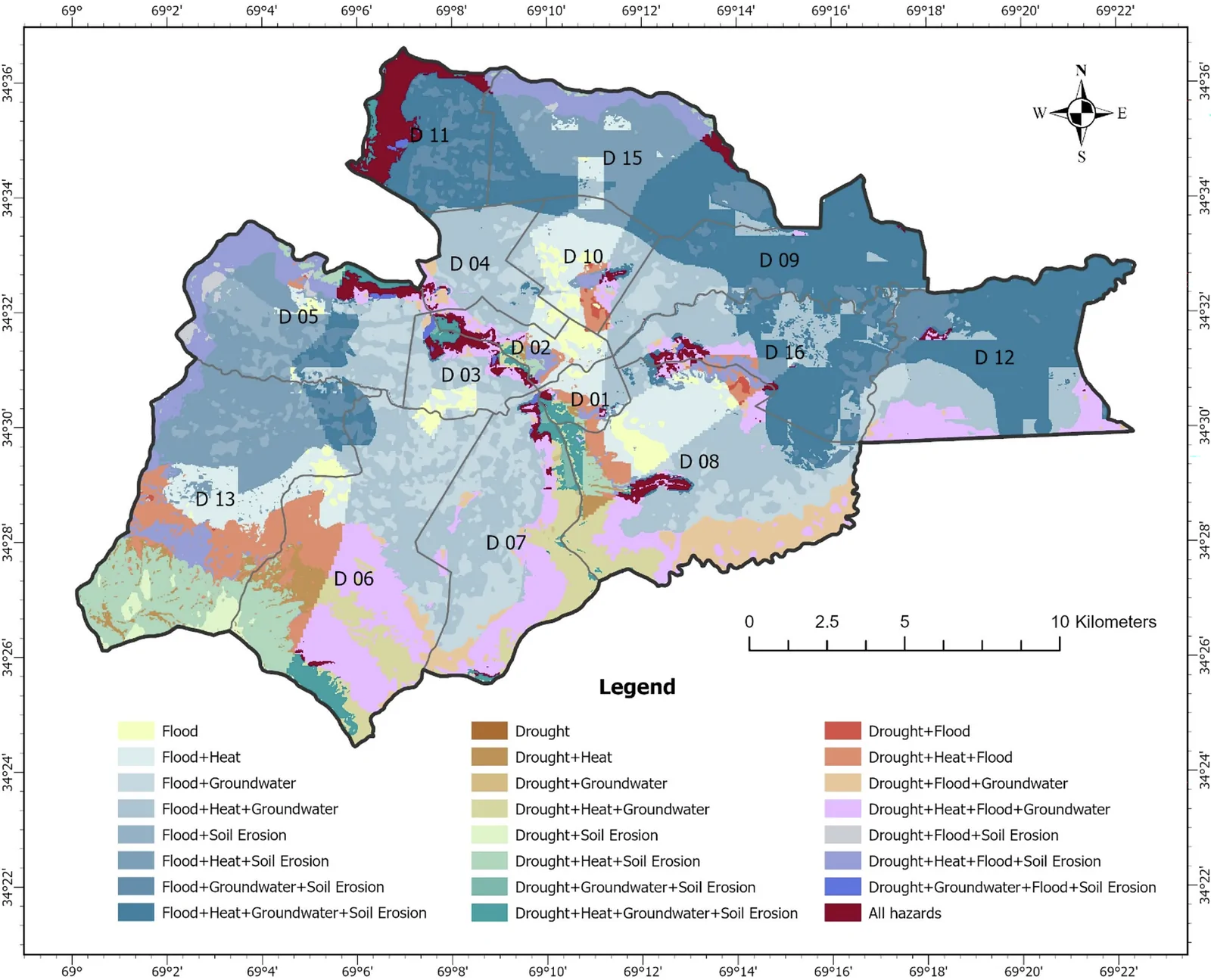
Types and zones of multi-hazard combinations (D01-D22 corresponds to Districts 1-22)

The multi-hazard assessment revealed notable spatial heterogeneity in hazard susceptibility across the study area. The assessment of multi-hazard zones (Figs. 5 and 6) shows that approximately 80% of the total area is exposed to at least three hazards, highlighting the pervasive nature of environmental hazards in the region. The spatial distribution is dominated by areas with a single hazard 1.8%, followed closely by zones with two overlapping hazards 18.2%. Critically, 44.3% of the area is affected by three or more hazards—32.9% with four and 2.8% with five hazards—designating these locations as priority zones for risk-sensitive planning (Fig. 5). The evaluation of the type of multi-hazard combinations (Fig. 7) offers further insights into the nature of these overlaps. Flooding emerged as the most spatially extensive individual hazard (90%), with frequent combinations including groundwater stress (8.47%), groundwater stress and heat hazard (17.79%), soil erosion and heat hazard (8.54%), groundwater stress, soil erosion and heat hazards (18.81%), and groundwater stress, drought and heat hazard (8.16%) (See Table 3).

**Table 3:** Multi-hazards combination and area coverage

No.	Unique combination	Area (km^2^)	Area (%)	No.	Unique combination	Area (km^2^)	Area (%)
0	No Hazard	0.00	0.00%	16	Drought	0.02	0.00%
1	Heat	0.00	0.00%	17	Drought+Heat	4.07	1.07%
2	Groundwater	0.00	0.00%	18	Drought+Groundwater	0.17	0.04%
3	Groundwater+Heat	0.00	0.00%	19	Drought+Groundwater+Heat	13.04	3.41%
4	Soil Erosion	0.00	0.00%	20	Drought+Soil Erosion	2.23	0.58%
5	Soil Erosion+Heat	0.00	0.00%	21	Drought+Soil Erosion+Heat	14.92	3.90%
6	Soil Erosion+Groundwater	0.00	0.00%	22	Drought+Soil Erosion+Groundwater	0.56	0.15%
7	Soil Erosion+Groundwater+Heat	0.00	0.00%	23	Drought+Soil Erosion+Groundwater+Heat	5.73	1.50%
8	Flood	6.79	1.78%	24	Drought+Flood	0.49	0.13%
9	Flood+Heat	24.07	6.30%	25	Drought+Flood+Heat	13.89	3.64%
10	Flood+Groundwater	32.36	8.47%	26	Drought+Flood+Groundwater	10.08	2.64%
11	Flood+Groundwater+Heat	67.97	17.79%	27	Drought+Flood+Groundwater+Heat	31.19	8.16%
12	Flood+Soil Erosion	6.01	1.57%	28	Drought+Flood+Soil Erosion	1.08	0.28%
13	Flood+Soil Erosion+Heat	32.61	8.54%	29	Drought+Flood+Soil Erosion+Heat	16.21	4.24%
14	Flood+Soil Erosion+Groundwater	15.29	4.00%	30	Drought+Flood+Soil Erosion+Groundwater	0.65	0.17%
15	Flood+Soil Erosion+Groundwater+Heat	71.85	18.81%	31	All hazards	10.80	2.83%

## Discussion

### Spatial Distribution of Individual Hazards

The first objective of this study was to develop detailed spatial hazard maps for five key environmental hazards affecting Kabul City: flood, heat, drought, groundwater stress, and soil erosion.

The flood hazard showed that nearly two-thirds of the study area lies within moderate to high-hazard zones, with central and eastern districts particularly affected. These findings correspond with those of Mushwani et al. (2024b) and Kourgialas and Karatzas (2011), reaffirming the critical role of inadequate drainage, low-lying topography, and unregulated urban development in exacerbating flood exposure. Importantly, we replaced flow accumulation with drainage density—following Gohil et al. (2024)—which improves the spatial representation of flood pathways, highlighting micro-watershed contributions in the hazard landscape. It is noteworthy that the results of the current study may differ from national flood hazard layers (Ikram et al., 2024). Such differences may arise from the selected parameters and the methodological approach adopted in this study, leading to variations in the spatial distribution and extent of flood hazards. The present study assesses flood hazard using selected spatial and environmental parameters within a multi-criteria framework, which provides valuable information for identifying hazard-prone areas at the local scale. However, this approach does not explicitly account for future climatic variability and hydrological processes (Iqbal et al., 2018). Therefore, the observed differences between the current findings and national-scale hazard datasets may also reflect variations in modeling frameworks, data inputs, and the representation of dynamic flood-generating processes. These findings underscore the necessity of integrating flood hazard data into urban planning frameworks. The spatial heterogeneity of flood hazard classes derived from this study not only highlights the urgency of adaptive flood risk management in highly vulnerable districts, including early warning systems and possible land use restrictions. It also offers a foundation for evidence-based NBS planning to enhance urban resilience against future flood events.

The heat hazard analysis identifies southern and northeastern districts as thermal hotspots, linked to sparse vegetation and dense impervious surfaces. The findings align with global and regional studies (Cheval et al., 2023; Khan et al., 2022) on urban heat islands and underscore the urgency for climate-sensitive planning. Referring to Raoufi et al. (2024), who have projected increasing temperature trends under future climate scenarios, highlighting growing thermal stress and climate vulnerability across the region. Projected increases in average, minimum, and maximum temperatures under different Representative Concentration Pathway scenarios further emphasize the likelihood of intensified heat-related challenges in the future. This complementary approach reinforces the importance of incorporating heat adaptation strategies into urban planning and climate resilience frameworks (Raoufi et al., 2024). The results support the strategic use of nature-based interventions tailored to the hazard profile of each district. Integrating heat hazard data into planning processes can enable authorities to identify priority zones, inform adaptive policy measures, and align infrastructure development with climate resilience objectives—especially in safeguarding public health and enhancing urban livability under future warming scenarios.

The drought hazard analysis shows higher vulnerability in southern and southwestern areas, in contrast to the flood-prone zones. This inverse spatial pattern confirms their mutual exclusivity, driven by climatic gradients and geomorphological contrasts, in line with Heydari Alamdarloo et al. (2020). From a risk management perspective, the spatial differentiation of drought hazard levels provides critical input for localized planning strategies. While the central and northern districts exhibit relatively low levels of hazard, the southern zones show significantly higher exposure, necessitating a more focused and proactive planning approach. These findings are also consistent with a recent study in the Kabul River Basin that highlighted increasing drought vulnerability and challenges to water resource sustainability under changing climatic conditions (Sediqi and Komori, 2024). The use of Standardized Runoff Index (SRI-3 and SRI-12) indicators under different climate scenarios demonstrated increasing variability in water availability and emphasized the importance of considering both short- and long-term hydrological dynamics for sustainable water resource management (Sediqi and Komori, 2024). However, some differences in spatial patterns may arise due to methodological variations, as the present study adopts a GIS-based environmental hazard assessment approach using spatial conditioning factors, whereas the cited study of Sediqi and Komori (2024) employed hydrological modeling (SWAT), runoff-based indicators, and future climate scenarios. These results offer a valuable foundation for the integration of NBS into drought risk reduction and urban planning frameworks. Particularly in high and very high hazard zones, the application of NBS can play a central role in enhancing ecological resilience, reducing drought impacts, and supporting sustainable land and water management practices. In moderate zones, preventive and adaptive planning informed by hazard levels can help mitigate the progression of risk, while low hazard areas offer opportunities for long-term resilience building.

The groundwater stress hazard analysis indicates elevated hazard in the western districts due to both depletion and nitrate contamination. These findings build on earlier research (Brati et al., 2019; Zaryab et al., 2022a), validating the dual focus on water quality and quantity. In this study, we conducted a spatially explicit groundwater stress assessment by integrating both groundwater quality and quantity indicators within a geospatial framework. This dual-dimensional approach, combining nitrate contamination levels and groundwater depth/depletion trends, provides a comprehensive perspective on aquifer vulnerability and sustainability across the study area. Integrating groundwater stress hazard assessments into broader urban and environmental planning frameworks will be crucial for enhancing water security, especially under increasing urban and climate pressures.

Lastly, soil erosion hazards remain generally very low to low, with moderate to high-hazard patches in sloped and vegetation-sparse areas. This pattern aligns with studies in similar terrains (Perera et al., 2020), confirming that steep slope and bare soil are primary erosion drivers. These findings provide a critical baseline for spatially targeted risk assessment and help guide NBS planning. Prioritizing interventions in moderate, high and very high-hazard zones can enhance landscape resilience, reduce sediment loads in downstream areas, and support sustainable urban and peri-urban land management strategies.

### Validation of Hazard Maps

Direct field-based validation of the hazard maps was not feasible due to the limited availability of comprehensive historical hazard inventories and georeferenced event records for the study area. Therefore, a qualitative plausibility assessment was conducted by comparing the spatial patterns of the produced hazard maps with findings reported in previous studies, official reports, and available hazard assessments.

For flood hazards, the identified high-hazard zones were compared with flood-prone areas reported by Mushwani et al. (2024a) and the Coalition for Disaster Resilient Infrastructure (Rudari, 2023b). Similarly, the spatial distribution of heat hazard hotspots and drought-prone areas was evaluated against the hazard patterns documented by Ranghieri et al. (2017) and the Coalition for Disaster Resilient Infrastructure (2023a). Groundwater stress results were assessed through comparison with the groundwater depletion and stress patterns reported by Hasani et al. (2025) and Zaryab et al. (2022b). In each case, the major spatial trends identified in the present study showed general agreement with the areas highlighted in the respective reference studies.

Although this comparison does not constitute a quantitative validation based on observed event inventories, it provides an independent plausibility check of the hazard maps and supports the reliability of the spatial patterns identified. Future studies could strengthen the validation process through the integration of georeferenced disaster records, historical event databases, remote-sensing-derived hazard inventories, and field observations as such data become available.

### Spatial Distribution of Multi-Hazards

The second objective sought to identify the spatial concurrence of multiple hazards. Prominent overlapped hazard clusters include flood–heat–groundwater stress and drought–heat–soil erosion, indicating the existence of ecological and infrastructural stress hotspots.

These results strongly support the findings of several studies (Skilodimou et al., 2019; Pourghasemi et al., 2019; Alikaei et al., 2023; Gohil et al., 2024; Summers et al., 2021; Morejón et al., 2025), which argue that single-hazard assessments are insufficient in complex urban settings. However, it is important to emphasize that the hazard maps generated in this study represent relative hazard susceptibility rather than probabilistic hazard assessments. The adopted MCDA-based approach identifies areas that are more likely to be exposed to specific hazards based on the selected conditioning factors and existing hazard information, but it does not quantify hazard magnitude, recurrence interval, exceedance probability, expected losses, or associated uncertainties. Similarly, the multi-hazard assessment reflects the spatial concurrence of hazard-prone areas rather than dynamic hazard interactions or cumulative risk. The resulting maps should be interpreted as indicators of relative hazard propensity and overlap that are suitable for preliminary screening, spatial prioritization, and strategic planning, particularly in data-scarce environments. By creating 24 distinct multi-hazard combinations, the study not only visualizes the spatial concurrence of multiple hazards but also captures the specific combinations of hazards affecting different locations, representing a novel contribution for Kabul and a replicable framework for other data-scarce cities. Flood-prone areas may benefit from measures such as water retention landscapes, wetland restoration, and river corridor management, while drought-prone zones may require interventions focused on water conservation, soil moisture enhancement, and drought-resilient vegetation. Collectively, these findings provide a foundational evidence base for the planning and prioritization of NBS. By identifying multi-hazard hotspots and examining dominant hazard combinations, the analysis enables stakeholders to better understand the spatial distribution of overlapping environmental hazards in rapidly developing urban and peri-urban areas of the Global South. Such insights are particularly valuable for urban planners and decision-makers seeking to implement spatially targeted, ecosystem-based resilience strategies. While the results highlight priority zones where such approaches may be most beneficial, the detailed design and specification of particular NBS fall beyond the scope of the present study. Instead, the spatial patterns identified here provide a strategic basis for guiding future planning processes and supporting the development of context-specific intervention measures.

### Future Research Needs

While this study suggests the potential of NBS as a means of hazard mitigation, prescribing specific interventions is beyond the scope of this paper. The delineation of hazard hotspots and multi-hazard combinations, however, provides the critical spatial intelligence necessary for targeted NBS planning—such as floodplain restoration, urban greening, or recharge area protection. Opportunities for future research thus include (i) integrating vulnerability and exposure datasets for comprehensive risk mapping, (ii) incorporating climate change projections to assess how hazard patterns may evolve under different scenarios, (iii) developing dynamic hazard models that account for temporal variability, seasonal changes, and interactions among hazards, rather than relying on predominantly static representations, (iv) comparing the MCDA-based framework applied in this study with machine learning and hybrid approaches (Somnath, 2023), such as Random Forest, Gradient Boosting, Support Vector Machines, and deep-learning-based spatial prediction methods, to evaluate differences in predictive performance, uncertainty, and interpretability (Somnath, 2025), (v) building upon the analysis here by designing future scenarios that consider NBS to address hazards, (vi) assessing the potential impacts of proposed NBS, for example through scenario modeling and cost–benefit analysis, and (vii) exploring opportunities for linking spatial hazard mapping with participatory urban planning tools and decision-making platforms, particularly in contexts like Kabul where institutional capacity and data availability remain limited.

## Conclusions

This study addressed the spatial distribution and concurrence of five major environmental hazards—flood, heat, drought, groundwater stress, and soil erosion—in Kabul City, a rapidly urbanizing and data-scarce metropolitan region. Using a GIS-based MCDA framework, individual hazard maps were developed and integrated to identify areas exposed to multiple environmental pressures. The results indicate that environmental hazards are widespread across the city, with more than 90% of the study area exposed to at least one hazard and distinct clusters of overlapping hazards concentrated in specific urban and peri-urban zones. Beyond these site-specific findings, the study makes three principal contributions. First, it provides one of the first integrated assessments of multiple environmental hazards in Kabul, generating a spatially explicit understanding of both individual hazards and their concurrence. Second, it demonstrates a practical methodological framework for multi-hazard assessment in data-constrained environments through the integration of heterogeneous environmental datasets within a GIS–MCDA approach. Third, by identifying 24 distinct multi-hazard combinations, the study moves beyond conventional single-hazard analyzes and provides a more comprehensive representation of the environmental pressures affecting rapidly growing cities. The findings also have important planning implications. The identified hazard hotspots and overlapping hazard zones provide a spatial basis for prioritizing interventions, supporting risk-sensitive land-use planning, and informing the strategic targeting of NBS. However, the results should be interpreted as relative hazard susceptibility patterns rather than validated predictions of hazard occurrence or risk, given the absence of quantitative validation datasets and the exclusion of exposure and vulnerability components. While the study focuses primarily on hazard characterization, it establishes a foundation for more comprehensive risk assessments that integrate socio-economic vulnerability, exposure, and adaptive capacity. Future research should further strengthen the framework through dynamic hazard modeling, climate change scenario analysis, quantitative validation, uncertainty assessment, and comparison with emerging machine-learning approaches. As cities across the Global South face increasing pressures from climate change, environmental degradation, and rapid urban expansion, integrated multi-hazard assessments can play an important role in supporting evidence-based urban resilience planning and risk governance. By providing a transparent and transferable framework for identifying priority areas of environmental concern, this study contributes to the broader goal of developing more adaptive, risk-informed, and resilient urban development pathways in data-scarce contexts.

### Critical Appraisal of Methods and Remaining Uncertainties

The methodological framework employed in this study relied heavily on GIS-based spatial analysis, supported by already defined rankings for each parameter of environmental hazards (Gohil et al., 2024; Mushwani et al., 2024b; Cheval et al., 2023); for individual hazards, different parameterizations are possible (Sharma and Kumar Rana, 2024). However, the modification of selected parameters (e.g., removal of population density from flood analysis) ensured hazard-centric prioritization but simultaneously removed a key proxy for exposure. While this improves hazard specificity, it limits direct risk estimation (Adem Esmail and Geneletti, 2018).

A limitation of this study is the integration of datasets with varying spatial resolutions and accuracies, which may introduce uncertainty despite resampling to a common 30 m grid. Additionally, the interpolation of point-based groundwater data may not fully capture local variability, potentially affecting the precision of the multi-hazard map.

Uncertainty is also associated with DEM-derived parameters, including elevation, slope, and drainage density, which may be influenced by DEM resolution and vertical accuracy, particularly in heterogeneous urban and peri-urban environments.

The temporal scope of input data varied widely across hazard types—LST was a single image from 2020, while precipitation data spanned a decade. This may have introduced temporal inconsistency in hazard representation. Moreover, differences between the present findings and Raoufi et al. (2024) may arise due to methodological and temporal differences, as the present study identifies spatial heat hazard patterns using Landsat-derived LST, whereas Raoufi employed statistical downscaling models (SDSM and LARS-WG) to evaluate long-term climate trends and future temperature projections.

Interpolation techniques (IDW, kriging, Thiessen polygons) were necessary due to the sparse and uneven distribution of field data, particularly for nitrate concentrations and groundwater depth and depletion. While methodologically sound, these introduce spatial uncertainty—especially in areas lacking adequate sampling coverage.

The soil erosion assessment represents relative erosion susceptibility rather than quantitative soil loss, as detailed rainfall erosivity, soil erodibility, and sediment transport measurements were not available for the study area.

The binary classification of hazard zones (i.e., moderate/high/very high = 1, others = 0) simplified the overlay analysis but also introduced a loss of granularity, especially in areas with moderate hazard levels that may still be significant under compound effects.

The applied methodology provides a replicable and scalable foundation for integrated urban hazard assessments, especially in contexts with similar data limitations. It also complements recent efforts to mainstream spatial analysis in disaster risk governance and urban resilience planning (Albert et al., 2021; Gohil et al., 2024; Hussain et al., 2023).

Although some degree of correlation may exist among environmental predictors (e.g., slope and elevation), the MCDA framework applied in this study is knowledge-driven rather than statistically inferred. Therefore, multicollinearity does not affect parameter estimation in the same manner as in regression-based models (Feizizadeh et al., 2014). Nevertheless, correlated variables may still introduce redundancy, which represents a limitation of the approach and should be considered in future studies (Arabameri et al., 2020; Pourghasemi et al., 2019).

An additional limitation relates to the restricted ability to conduct field verification within the study area. Due to the prevailing political and security conditions in Afghanistan during the study period, in-situ field visits and primary data collection were not feasible. As a result, the analysis relied predominantly on secondary sources and globally available datasets. While these datasets are widely used in regional-scale environmental and hazard assessments, the absence of field validation may introduce additional uncertainty in the spatial representation of certain parameters. Future studies incorporating ground-based observations and local monitoring data would help further refine and validate the results presented in this study.

Additional sources of uncertainty relate to spatial autocorrelation, scale dependency, and the potential influence of the selected analysis resolution. Although a raster-based framework was employed to reduce aggregation effects associated with administrative units, the results may still be sensitive to spatial scale and dataset resolution. Future studies should investigate these effects through formal sensitivity analyzes, spatial autocorrelation measures, and multi-scale assessments.

## Supplementary information

Supplementary information

## Data Availability

Data will be made available on request.
