# Supplementary material for: Unveiling Multi-Hazard Hotspots in Kabul: An Integrated Geospatial Assessment in a Data-Scarce Urban Environment
Source: Environ Manage. 2026 Sep 3;76(9):300. doi: 10.1007/s00267-026-02588-w (PMC13541899; doi:10.1007/s00267-026-02588-w)
Supplement: Supplementary file 1 [file 267_2026_2588_MOESM1_ESM.docx]

**Supplementary Information (SI)**

**Article title:** Unveiling Multi-Hazard Hotspots in Kabul: An Integrated Geospatial Assessment in a Data-Scarce Urban Environment

**Journal name:** Environmental Management

**Author names:** Maisam Rafiee*, Mohammad Reza Ansari, Daniela Kempa, Tinka Kuhn, Blal Adem Esmail, Lisa Biber-Freudenberger, Christian Albert

**Corresponding author:** Maisam Rafiee

**Affiliation:** Institute of Environmental Planning, Leibniz University Hannover, Herrenhäuserstr.2, 30419 Hannover, Germany

**Email address:** rafiee@umwelt.uni-hannover.de

Table 4 LULC Confusion Matrix

| Class Value | Agricultural Land | Vegetation Cover | Built-up | Bare Land | Water Body | Total | U_Accuracy | Kappa |
| --- | --- | --- | --- | --- | --- | --- | --- | --- |
| Agricultural Land | 10 | 0 | 0 | 0 | 0 | 10 | 1 | 0 |
| Vegetation Cover | 0 | 5 | 0 | 5 | 0 | 10 | 0.5 | 0 |
| Built-up | 0 | 1 | 9 | 0 | 0 | 10 | 0.9 | 0 |
| Bare Land | 0 | 0 | 0 | 10 | 0 | 10 | 1 | 0 |
| Water Body | 0 | 0 | 0 | 0 | 10 | 10 | 1 | 0 |
| Total | 10 | 6 | 9 | 15 | 10 | 50 | 0 | 0 |
| P_Accuracy | 1 | 0.833333 | 1 | 0.666667 | 1 | 0 | 0.88 | 0 |
| Kappa | 0 | 0 | 0 | 0 | 0 | 0 | 0 | 0.85 |

*Table 5a The parameters, their classification and rating for flood hazard assessment*

| *Parameters* | *Classification* | | *Rank (a)* | *Hazard rating* |
| --- | --- | --- | --- | --- |
| Slope (degree) | 0 | 4.04458 | 5 | Very high |
|  | 4.04458 | 10.3361 | 4 | High |
|  | 10.3361 | 18.2006 | 3 | Moderate |
|  | 18.2006 | 26.9639 | 2 | Low |
|  | 26.9639 | 57.2982 | 1 | Very low |
| Drainage Density (km/km2) | 0 | 0.63746 | 1 | Very low |
|  | 0.63746 | 1.29263 | 2 | Low |
|  | 1.29263 | 1.89468 | 3 | Moderate |
|  | 1.89468 | 2.6738 | 4 | High |
|  | 2.6738 | 4.51536 | 5 | Very high |
| Precipitation 2013-2022 (mm) | 360.977 | 380.514 | 1 | Very low |
|  | 380.514 | 391.104 | 2 | Low |
|  | 391.104 | 400.239 | 3 | Moderate |
|  | 400.239 | 409.905 | 4 | High |
|  | 409.905 | 431.034 | 5 | Very high |
| Soil type | Glaciers/Open water | | 5 | Very high |
|  | Solonetz |  | 4 | High |
|  | Nitisols |  | 3 | Moderate |
|  | Arenosols | | 2 | Low |
|  |  |  |  |  |
| LULC | Agricultural Land | | 1 | Very low |
|  | Vegetation Cover | | 2 | Low |
|  | Built-up | | 3 | Moderate |
|  | Bare Land | | 4 | High |
|  | Water Body | | 5 | Very high |
| Elevation (m) | 1760 | 1846 | 5 | Very high |
|  | 1846 | 1961 | 4 | High |
|  | 1961 | 2147 | 3 | Moderate |
|  | 2147 | 2436 | 2 | Low |
|  | 2436 | 3003 | 1 | Very low |

*Table 5b Scaling of Flood hazard criterion based on pairwise comparison*

| Criteria | Slope | DD | Precip. | Soil type | LULC | Elevation |
| --- | --- | --- | --- | --- | --- | --- |
| Slope | 1 | 0.5 | 0.333333 | 2 | 3 | 0.333333 |
| DD | 2 | 1 | 2 | 4 | 5 | 0.5 |
| Precip. | 3 | 0.5 | 1 | 3 | 4 | 0.333333 |
| Soil type | 0.5 | 0.25 | 0.333333 | 1 | 3 | 0.333333 |
| LULC | 0.333333 | 0.2 | 0.25 | 0.333333 | 1 | 0.25 |
| Elevation | 3 | 2 | 3 | 3 | 4 | 1 |
| Sum | 9.833333 | 4.45 | 6.916667 | 13.33333 | 20 | 2.75 |

*Table 5c Normalization of scores, estimation of weights and consistency ratio (CR)*

| Criteria | Slope | DD | Precip. | Soil type | LULC | Elevation | Average | Lampda | Consistency Index | Random Index | **CR** |
| --- | --- | --- | --- | --- | --- | --- | --- | --- | --- | --- | --- |
| Slope | 0.101695 | 0.11 | 0.048193 | 0.15 | 0.15 | 0.121212 | 0.11 | 6.265082 | 0.102854 | 1.24 | **0.08295** |
| DD | 0.20339 | 0.224719 | 0.289157 | 0.3 | 0.25 | 0.181818 | 0.24 | 6.508338 |  |  |  |
| Precip. | 0.305085 | 0.11236 | 0.144578 | 0.225 | 0.2 | 0.121212 | 0.18 | 6.469748 |  |  |  |
| Soil type | 0.050847 | 0.05618 | 0.048193 | 0.075 | 0.15 | 0.121212 | 0.08 | 6.13542 |  |  |  |
| LULC | 0.033898 | 0.044944 | 0.036145 | 0.025 | 0.05 | 0.090909 | 0.05 | 6.183641 |  |  |  |
| Elevation | 0.305085 | 0.449438 | 0.433735 | 0.225 | 0.2 | 0.363636 | 0.33 | 6.51427 |  |  |  |

***Fig 8*** *Thematic maps illustrating each parameter used in flood hazard assessment*

**LST detailed process:**

The stepwise process was implemented in ArcGIS Pro 3.4.3 using a custom-built model to automate the workflow. the process includes the following steps:

(i) Top of Atmospheric Spectral Radiance (TOA): The digital number (DN) values from Band 10 were converted to spectral radiance using the radiometric rescaling factors provided in the image metadata:

$L\lambda=ML*Qcal+AL$	(1)

In which, Lλ is the spectral radiance (W/m2×sr × µm), ML is multiplicative scaling factor of radiance for the band, AL is additive scaling factor of radiance for band and QCAL is the pixel value in digital number.

(ii) Brightness Temperature (BT): The spectral radiance was then transformed into brightness temperature using the thermal constants K1 and K2:

$T=\left( \frac{K2}{ln\left( \frac{K1}{L\lambda} \right)}+1 \right)-273.15$	(2)

In which, T is TOA Brightness temperature, K1 is the thermal conversion constant from metadata, K2 is the thermal conversion constant from meta data for different thermal bands.

(iii) Normalized Difference Vegetation Index (NDVI): NDVI was calculated from the Red (Band 4) and NIR (Band 5) bands to evaluate vegetation cover:

$NDVI=\frac{Band5-Band4}{Band5+Band4}$	(3)

(iv) Proportion of Vegetation (PV): Based on the NDVI values, the proportion of vegetation was derived:

$P\nu=\left( \frac{NDVI-NDVImin}{NDVImax-NDVImin} \right)^{2}$	(4)

(v) Surface Emissivity value (ε): The emissivity, which affects how different surfaces emit thermal radiation, was estimated as:

$\varepsilon_{\nu}=0.004* P\nu+0.986$	(5)

(vi) Land Surface Temperature (LST): Finally, the LST was computed by correcting the brightness temperature using surface emissivity:

$LST=(\frac{BT}{1+\left( \lambda*\frac{BT}{p} \right)*Ln\left( \varepsilon\right)})$	(6)

In which, h is Planck’s constant = 6.626∗10−34 J s, c is velocity of light = 2.998∗10^8^ m/s and s is Boltzmann constant = 1.38 ∗ 10^−23^ J/K, p = h*c/s =1.438 × 10^-2^ m, K=1438 μm K, λ wave length = 10.895 μm.

*Table 6 Land Surface Temperature (LST) classification and rating*

| *Parameters* | *Classification* | | *Rank (a)* | *Hazard rating* |
| --- | --- | --- | --- | --- |
| Land Surface Temprature (LST) | 25.979 | 34.092 | 1 | Very low |
|  | 34.093 | 37.077 | 2 | Low |
|  | 37.078 | 39.969 | 3 | Moderate |
|  | 39.970 | 43.513 | 4 | High |
|  | 43.514 | 49.763 | 5 | Very high |

***Fig 9*** *Thematic maps of processed factors for Land Surface Temperature (LST) calculation*

*Table 7a The parameters, their classification and rating for drought hazard assessment*

| *Parameters* | *Classification* | | *Rank (a)* | *Hazard rating* |
| --- | --- | --- | --- | --- |
| Slope (degree) | 0.000 | 4.045 | 1 | Very low |
|  | 4.045 | 10.336 | 2 | Low |
|  | 10.336 | 18.201 | 3 | Moderate |
|  | 18.201 | 26.964 | 4 | High |
|  | 26.964 | 57.298 | 5 | Very high |
| Precipitation 2013-2022 (mm) | 360.977 | 380.514 | 5 | Very high |
|  | 380.514 | 391.104 | 4 | High |
|  | 391.104 | 400.239 | 3 | Moderate |
|  | 400.239 | 409.905 | 2 | Low |
|  | 409.905 | 431.034 | 1 | Very low |
| Soil type |  | | 5 | Very high |
|  | Arenosols | | 4 | High |
|  | Nitisols | | 3 | Moderate |
|  | Solonetz | | 2 | Low |
|  | Glaciers/Open water | | 1 | Very low |
| LULC | Water Body | | 1 | Very low |
|  | Vegetation Cover | | 2 | Low |
|  | Agricultural Land | | 3 | Moderate |
|  | Built-up | | 4 | High |
|  | Bare Land | | 5 | Very high |
| Elevation (m) | 1760 | 1846 | 1 | Very low |
|  | 1846 | 1961 | 2 | Low |
|  | 1961 | 2147 | 3 | Moderate |
|  | 2147 | 2436 | 4 | High |
|  | 2436 | 3003 | 5 | Very high |
| Drainage Density (km/sqkm) | 0 | 0.637462 | 5 | Very high |
|  | 0.63746 | 1.292632 | 4 | High |
|  | 1.29263 | 1.894679 | 3 | Moderate |
|  | 1.89468 | 2.6738 | 2 | Low |
|  | 2.6738 | 4.515357 | 1 | Very low |

*Table 7b Scaling of drought hazard criterion based on pairwise comparison*

| Criteria | Slope | DD | Precip. | Soil type | LULC | Elevation |
| --- | --- | --- | --- | --- | --- | --- |
| Slope | 1 | 0.25 | 0.3333 | 0.3333 | 2 | 0.2 |
| DD | 4 | 1 | 2 | 4 | 3 | 0.5 |
| Precip. | 3 | 0.5 | 1 | 3 | 4 | 0.3333 |
| Soil type | 3 | 0.25 | 0.3333 | 1 | 2 | 0.25 |
| LULC | 0.5 | 0.3333 | 0.25 | 0.5 | 1 | 0.25 |
| Elevation | 5 | 2 | 3 | 4 | 4 | 1 |
| Sum | 16.5 | 4.3333 | 6.9166 | 12.8333 | 16 | 2.5333 |

*Table 7c Normalization of scores, estimation of weights and consistency ratio (CR)*

| Criteria | Slope | DD | Precip. | Soil type | LULC | Elevation | Average | Lampda | Consistency Index | Random Index | **CR** |
| --- | --- | --- | --- | --- | --- | --- | --- | --- | --- | --- | --- |
| Slope | 0.0606 | 0.0577 | 0.0482 | 0.0260 | 0.1250 | 0.0789 | 0.07 | 6.1248 | 0.1193 | 1.24 | **0.09624** |
| DD | 0.2424 | 0.2308 | 0.2892 | 0.3117 | 0.1875 | 0.1974 | 0.24 | 6.5967 |  |  |  |
| Precip. | 0.1818 | 0.1154 | 0.1446 | 0.2338 | 0.2500 | 0.1316 | 0.18 | 6.4666 |  |  |  |
| Soil type | 0.1818 | 0.0577 | 0.0482 | 0.0779 | 0.1250 | 0.0987 | 0.10 | 6.3148 |  |  |  |
| LULC | 0.0303 | 0.0769 | 0.0361 | 0.0390 | 0.0625 | 0.0987 | 0.06 | 6.1879 |  |  |  |
| Elevation | 0.3030 | 0.4615 | 0.4337 | 0.3117 | 0.2500 | 0.3947 | 0.36 | 6.4775 |  |  |  |

***Fig 10*** *Thematic maps illustrating each parameter used in drought hazard assessment*

*Table 8a The parameters, their classification and rating for groundwater stress hazard assessment*

| *Parameters* | | *Classification* | | *Rank (a)* | *Hazard rating* |
| --- | --- | --- | --- | --- | --- |
| Groundwater Quantity | GroundWater Depletion (m) | 0.000 | 13.000 | 1 | Very low |
|  |  | 13.001 | 26.000 | 2 | Low |
|  |  | 26.001 | 38.000 | 3 | Moderate |
|  |  | 38.001 | 51.000 | 4 | High |
|  |  | 51.001 | 65.000 | 5 | Very high |
|  | GroundWater Depth (m) | 13.750 | 24.394 | 1 | Very low |
|  |  | 24.394 | 35.491 | 2 | Low |
|  |  | 35.491 | 47.494 | 3 | Moderate |
|  |  | 47.494 | 60.176 | 4 | High |
|  |  | 60.176 | 71.500 | 5 | Very high |
| Groundwater Quality | NO3 (mg/lit) | 0 | 5 | 1 | Very low |
|  |  | 5 | 15 | 2 | Low |
|  |  | 15 | 25 | 3 | Moderate |
|  |  | 25 | 40 | 5 | Very high |
|  |  | 40 | 50 | 5 | Very high |

*Table 8b Scaling of groundwater stress criterion based on pairwise comparison*

| Criteria | GW_Deplation | GW_Depth | GW_Quality |
| --- | --- | --- | --- |
| GW_Deplation | 1 | 0.5 | 0.2 |
| GW_Depth | 2 | 1 | 0.2 |
| GW_Quality | 5 | 5 | 1 |
| Sum | 8 | 6.5 | 1.4 |

*Table 8c Normalization of scores, estimation of weights and consistency ratio (CR)*

| Criteria | GW_Deplation | GW_Depth | GW_Quality | Average | Lampda | Consistency Index | Random Index | **CR** |
| --- | --- | --- | --- | --- | --- | --- | --- | --- |
| GW_Deplation | 0.1250 | 0.0769 | 0.1429 | 0.1149 | 3.0159 | 0.0570 | 0.58 | **0.09828** |
| GW_Depth | 0.2500 | 0.1538 | 0.1429 | 0.1822 | 3.0327 |  |  |  |
| GW_Quality | 0.6250 | 0.7692 | 0.7143 | 0.7028 | 3.1140 |  |  |  |

***Fig 11*** *Thematic maps illustrating each parameter used in groundwater stress hazard assessment, Groundwater quantity is the overlapping result of groundwater depth and depletion*

*Table 9a The parameters, their classification and rating for soil erosion hazard assessment*

| *Parameters* | *Classification* | | *Rank (a)* | *Hazard rating* |
| --- | --- | --- | --- | --- |
| LULC | Built-up | | 1 | Very low |
|  | Bare Land | | 2 | Low |
|  | Vegetation Cover | | 3 | Moderate |
|  | Agricultural Land | | 4 | High |
|  | Water Body | | 5 | Very high |
| Soil type | Glaciers/Open water | | 5 | Very high |
|  | Solonetz | | 4 | High |
|  | Nitisols | | 3 | Moderate |
|  | Arenosols | | 2 | Low |
| Precipitation 2013-2022 (mm) | 360.977 | 380.514 | 1 | Very low |
|  | 380.514 | 391.104 | 2 | Low |
|  | 391.104 | 400.239 | 3 | Moderate |
|  | 400.239 | 409.905 | 4 | High |
|  | 409.905 | 431.034 | 5 | Very high |
| Slope (degree) | 0.000 | 4.045 | 1 | Very low |
|  | 4.045 | 10.336 | 2 | Low |
|  | 10.336 | 18.201 | 3 | Moderate |
|  | 18.201 | 26.964 | 4 | High |
|  | 26.964 | 57.298 | 5 | Very high |
| Elevation (m) | 1760 | 1846 | 1 | Very low |
|  | 1846 | 1961 | 2 | Low |
|  | 1961 | 2147 | 3 | Moderate |
|  | 2147 | 2436 | 4 | High |
|  | 2436 | 3003 | 5 | Very high |

*Table 9b Scaling of soil erosion hazard criterion based on pairwise comparison*

| Criteria | LULC | Soil type | Precip. | Slope | Elevation |
| --- | --- | --- | --- | --- | --- |
| LULC | 1 | 0.333333 | 0.2 | 0.2 | 2 |
| Soil type | 3 | 1 | 0.333333 | 0.333333 | 3 |
| Precip. | 5 | 3 | 1 | 2 | 5 |
| Slope | 5 | 3 | 0.5 | 1 | 3 |
| Elevation | 0.5 | 0.333333 | 0.2 | 0.333333 | 1 |
| Sum | 14.5 | 7.666667 | 2.233333 | 3.866667 | 14 |

*Table 9c Normalization of scores, estimation of weights and consistency ratio (CR)*

| Criteria | LULC | Soil type | Precip. | Slope | Elevation | Average | Lampda | Consistency Index | Random Index | **CR** |
| --- | --- | --- | --- | --- | --- | --- | --- | --- | --- | --- |
| LULC | 0.06897 | 0.04348 | 0.08955 | 0.05172 | 0.14286 | 0.08 | 5.0620 | 0.1077 | 1.12 | **0.09612** |
| Soil type | 0.20690 | 0.13043 | 0.14925 | 0.08621 | 0.21429 | 0.16 | 5.2295 |  |  |  |
| Precip. | 0.34483 | 0.39130 | 0.44776 | 0.51724 | 0.35714 | 0.41 | 5.2928 |  |  |  |
| Slope | 0.34483 | 0.39130 | 0.22388 | 0.25862 | 0.21429 | 0.29 | 5.4306 |  |  |  |
| Elevation | 0.03448 | 0.04348 | 0.08955 | 0.08621 | 0.07143 | 0.07 | 5.1518 |  |  |  |

***Fig 12*** *Thematic maps illustrating each parameter used in soil erosion hazard assessment*
